# *QuickStats*: Percentage* of Adults Aged ≥18 Years With Fair or Poor Health,^†^ by Urbanization Level^§^ and Age Group — National Health Interview Survey, United States, 2019^¶^

**DOI:** 10.15585/mmwr.mm7030a3

**Published:** 2021-07-30

**Figure:**
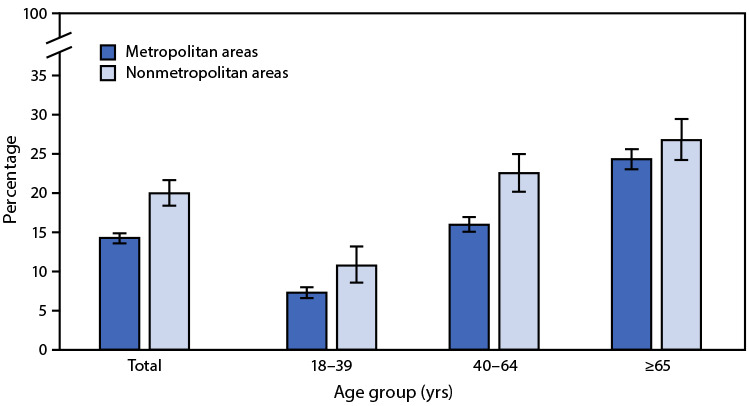
In 2019, the percentage of adults aged ≥18 years reported to be in fair or poor health was higher among those living in nonmetropolitan areas (20.3%) than among those living in metropolitan areas (14.5%). Percentages in fair or poor health were higher in nonmetropolitan areas for those aged 18–39 years (10.9% versus 7.4%) and 40–64 years (22.9% versus 16.2%), but the difference by urbanization level did not reach statistical significance for adults aged ≥65 years (27.2% versus 24.7%). The percentage reporting fair or poor health increased with age in both nonmetropolitan and metropolitan areas.

**Source:** National Center for Health Statistics, National Health Interview Survey, 2019. https://www.cdc.gov/nchs/nhis.htm

